# Layered vulnerabilities in young adults with major depressive disorder: personality, coping, and cognition

**DOI:** 10.3389/fpsyt.2026.1727879

**Published:** 2026-02-24

**Authors:** Gabrielle Wann Nii Tay, Glenn Tze Yi Tay, Cyrus Su Hui Ho

**Affiliations:** 1 Department of Psychological Medicine, Yong Loo Lin School of Medicine, National University of Singapore, Singapore, Singapore; 2 Department of Psychological Medicine, National University Hospital, Singapore, Singapore, Singapore

**Keywords:** coping, early detection, major depressive disorder, personality, screening, subjective cognition, young adults

## Abstract

**Objectives:**

Major depressive disorder (MDD) is a leading cause of disability worldwide, with peak incidence in young adulthood. Personality traits, coping styles, and cognitive functioning are established vulnerabilities, yet few studies have examined these dimensions together in young Asian adults. This study investigates whether personality, coping, and cognition function as layered vulnerabilities for MDD and whether their integration enhances diagnostic accuracy and identifies intervention targets, specifically in young Singaporean adults.

**Methods:**

In an exploratory case–control study, 36 patients with MDD and 36 matched healthy controls aged 21–29 years completed validated measures of personality traits, coping strategies, and cognition. Analyses included group comparisons, correlational analyses, and hierarchical regression models with false discovery rate correction.

**Results:**

Compared with controls, patients scored lower in emotional stability, conscientiousness, extraversion, and agreeableness; relied more on maladaptive coping; and reported more perceived cognitive deficits (all *q* < 0.05). Emotional stability and subjective cognition were the strongest predictors of MDD status: adding emotional stability to the baseline demographic model markedly improved diagnostic accuracy (*AUC* = 0.920, *ΔAUC* = 0.170, *p* < 0.001), while subjective cognition (but not objective performance) provided a modest additional increase (*AUC* = 0.954, *p* < 0.001). In the patient subsample (N = 36), maladaptive coping also significantly predicted depression severity.

**Conclusions:**

Personality, coping, and subjective cognition reflect layered vulnerabilities for MDD in young adults. Emotional stability emerged as the most impactful distal predictor, while perceived cognitive deficits provided a proximal, state-dependent marker, modestly enhancing diagnostic accuracy. Maladaptive coping related to symptom severity, highlighting its role as a potential intervention target. These findings illustrate how distal, semi-malleable, and proximal factors can inform early detection and targeted interventions.

## 1 Introduction

Major depressive disorder (MDD), or depression, is a leading cause of global disability, affecting more than 264 million people worldwide and generating profound social and economic costs (1). MDD incidence peaks in adolescence and young adulthood, with those aged 18–29 years at greatest risk (2, 3). This heightened vulnerability stems from the unique developmental challenges of this life stage — identity formation, academic and vocational pressures, shifting social roles, and evolving interpersonal relationships — that converge to increase susceptibility to depression and related psychiatric disorders. Early depression onset in young adults is strongly associated with more severe long-term outcomes, particularly when combined with high levels of early life stress (4), highlighting the need for early identification and intervention.

In Singapore, the 2016 Singapore Mental Health Study reported a lifetime prevalence of 6.3% for MDD, with the highest rates observed among young adults (5). Large-scale surveys across Asia including China, Indonesia, and other Southeast Asian countries, corroborate these findings, showing elevated prevalence in this demographic (6–8). However, much of depression research has focused on Western, middle-aged populations, leaving young Asian adults underrepresented (9, 10).

Depression vulnerability is shaped by developmental stage, environmental factors, and cultural norms. Personality traits, coping strategies, and cognitive functioning all contribute to depression risk, and their relative importance may differ across age groups and cultural contexts (11, 12). Although the epidemiological burden is clear, the personality, coping, and cognitive profiles that confer vulnerability in this population remain poorly characterized compared with non-Asian populations (9, 10). The present study therefore aims to examine these dimensions of depression vulnerability collectively in young Singaporean adults.

Personality traits are a well-established dimension of vulnerability for depression. Within the Big Five framework, neuroticism consistently predicts both the onset and persistence of depression while conscientiousness and extraversion confer protection through self-regulation and social engagement (13, 14). These associations may be particularly pronounced in young adults, where neuroticism peaks and depression risk is also the highest (2, 15). Although well documented in Western populations, evidence in Asian cohorts is still emerging, with recent studies in Singapore suggesting that low emotional stability is a robust marker of depression (5, 16).

Beyond the Big Five, other theoretical frameworks have also examined the relationship between personality and depression. Cloninger’s Psychobiological Model of Temperament and Character identifies biologically-influenced temperament dimensions (e.g., harm avoidance, reward dependence) and character traits (e.g., self-directedness, cooperativeness) associated with depression vulnerability (17), a relationship confirmed by a recent meta-analysis across clinical and non-clinical populations (18). Cognitive vulnerability theories similarly suggest that stable, trait like patterns of maladaptive thinking, such as a ruminative response style, predispose individuals to the onset and recurrence of depression (19). While these perspectives provide complementary insights, the Big Five remains the most widely validated and cross-culturally applied model (14), with strong evidence linking its traits to depression across the lifespan (20). Recent longitudinal research in young adult cohorts has also shown that higher neuroticism and lower extraversion predict the onset of depressive disorders, underscoring the relevance of the Big Five for studying vulnerability in young adults (21). While personality-depression associations appear robust across cultures (14), most evidence comes from Western samples. Whether personality vulnerability profiles differ between Western and Asian young adults remains unclear, as direct comparative studies are limited.

Building on personality traits, coping styles constitute another key dimension of vulnerability for depression. Maladaptive coping strategies, like self-blame and rumination, are consistently associated with greater depressive symptoms, whereas adaptive coping strategies like problem-solving and positive reframing promote resilience (22, 23). While most evidence comes from Western populations, emerging research in Asian samples suggests a similar pattern, with maladaptive coping increasing depression risk and adaptive strategies providing protection (16, 24). Common stressors in young adulthood, including career uncertainty and relational instability, may further amplify these effects, highlighting the importance of examining coping in young adults.

Cognitive functioning forms a third dimension of vulnerability for depression, often manifesting as difficulties in concentration, memory, and decision-making. Cognitive impairments are frequently reported by individuals with MDD, and have been linked to heightened cognitive load in modern environments characterized by pervasive multitasking and high informational demands (25, 26). A notable divergence exists between subjective and objective cognition: perceived cognitive deficits correlate strongly with depressive symptoms and everyday functioning, whereas objective test performance shows weaker associations (27, 28). In young adults, subjective cognition complaints may be particularly salient, as perceived deficits can undermine academic achievement, self-confidence, and daily functioning, all of which are closely tied to depression severity. Longitudinal evidence confirms a bidirectional relationship: worsening depressive symptoms predict increases in subjective cognitive complaints, and vice versa (29). Emerging evidence in Asian samples suggest that subjective cognitive deficits predict impairment even when objective deficits are minimal (30, 31), although such research remain limited compared to Western populations.

Together, personality, coping, and cognition form a layered model of depression vulnerability. Personality traits represent stable, trait-based predispositions that shape broad patterns of emotional reactivity and regulation (13, 14). Coping strategies are influenced by enduring traits but also reflect situation-specific, state-like responses to stress, making them partly malleable and a promising target for intervention (22, 23). Cognitive behavioral therapy (CBT), for instance, has been shown to enhance adaptive coping and reduce depressive symptoms beyond pharmacotherapy alone, illustrating the clinical relevance of targeting coping strategies (32). Cognitive functioning, particularly subjective cognition, fluctuates with mood and symptom severity (28). This layered model aligns with contemporary frameworks of psychopathology, such as the trait activation theory and diathesis–stress models, which emphasize how enduring vulnerabilities interact with situational stressors to produce depressive outcomes (33, 34).

Despite the theoretical and clinical importance of this layered framework, studies that simultaneously examine personality, coping, and cognition in young Asian adults with depression remain limited. This gap is particularly notable given that cultural context may influence how depression vulnerability manifests and is expressed. For instance, in Asian contexts, where stigma and cultural norms often limit the sharing and expression of emotional distress, young adults may present with somatic, cognitive or behavioral symptoms rather than directly reporting mood disturbances (35, 36). In such contexts, cognitive complaints (e.g., poor concentration, memory difficulties) and observable coping patterns may serve as more culturally acceptable entry points for assessment than direct inquiry about mood symptoms (37). While our study does not directly measure cultural variables, this context underscores the practical value of examining personality, coping, and cognitive markers in young Singaporean adults.

Our earlier work demonstrated that low emotional stability and maladaptive coping were significant markers of MDD in Singaporean adults (16). Building on this foundation, the present study investigates whether personality traits, coping strategies, and cognitive functioning can jointly differentiate young adults (aged 21–29 years) with MDD from their healthy peers. We examine the associations within and between these dimensions, their relationship to depression severity, and whether combining them improves the accuracy of predicting MDD status. By focusing on young Singaporean adults, this study aims to provide preliminary insights into developmental and contextually relevant depression vulnerability markers, which may inform early detection strategies and the development of targeted interventions to improve treatment outcomes in this population.

## 2 Methods

### 2.1 Participants

A total of 36 patients with major depressive disorder (MDD) and 36 healthy controls (HCs) were recruited for this case-control study. The MDD group comprised 22 women and 14 men, while the HC group included 24 women and 12 men. All participants were aged between 21 and 29 years and English-speaking.

Patients were recruited from outpatient psychiatric clinics at a university hospital in Singapore, where they had received a diagnosis of MDD by a psychiatrist according to Diagnostic and Statistical Manual of Mental Disorders, Fifth Edition (DSM-5) criteria. HCs were recruited from the community and were matched to patients by sex, age (+/- 5), ethnicity, and years of education. Individuals with a history of psychiatric or neurological disorders, and conditions that could affect the central nervous system, including cerebrovascular disease, respiratory disease, hepatic disease, kidney disease, cancer, epilepsy, or intellectual disability, were not eligible for participation.

Sociodemographic information including age, sex, ethnicity, and highest education level was collected from all participants using self-administered questionnaires. Healthy controls were also asked about family history of psychiatric disorders. For patients with MDD, clinical information was obtained through self-administered questionnaires, including years since diagnosis, history of self-harm and suicide attempts, family history of psychiatric illness, duration of psychiatric medication use, and current fluoxetine-equivalent antidepressant dosages.

All participants provided written informed consent after receiving a full explanation of study procedures. The study was conducted in accordance with the Declaration of Helsinki and the Belmont Report. Ethical approval was obtained from the Domain Specific Review Board of the National Healthcare Group, Singapore (protocol number 2022/00164) and the Institutional Review Board of the National University of Singapore (reference number NUS-IRB-2022-259).

### 2.2 Measures

#### 2.2.1 Hamilton rating scale for depression

Depression severity was assessed using the 17-item Hamilton Depression Rating Scale (HAM-D-17), a clinician-administered instrument that evaluates depressive symptoms over the past 7 days (38). Items are scored on a 4-point scale based on the assessment and judgment of the clinician derived from the information elicited from the patient, including nonverbal cues. Severity categories are defined as mild (8-16), moderate (17-23), and severe (≥ 24). The HAM-D-17 items can be grouped into five domain subscales: insomnia, anxiety, somatic, melancholia, and response-based subdomains. The HAM-D-17 demonstrated good internal consistency in this sample (Cronbach’s alpha *α* = 0.85), consistent with previous validation studies.

#### 2.2.2 Ten-item personality inventory

The Ten-Item Personality Inventory (TIPI) is a brief 10-item self-report measure of Big Five personality dimensions: extraversion, agreeableness, conscientiousness, emotional stability (reverse-scored neuroticism), and openness to experience. Items are rated on a 7-point Likert scale from 1 (disagree strongly) to 7 (agree strongly). Each personality dimension is scored by calculating the mean of two items, one of which is reverse-coded. This yields dimension scores ranging from 1 to 7, with higher scores indicating greater levels of each trait. The TIPI is designed to be brief, with previous research showing it provides reliable personality assessment despite its short length (39), and it demonstrated acceptable internal consistency in this sample (Cronbach’s alpha *α* = 0.72).

#### 2.2.3 Brief coping orientation to problems experienced

The Brief COPE is a validated 28-item self-report questionnaire assessing coping strategies across 14 domains (2 items each): active coping, planning, positive reframing, acceptance, humor, religion, emotional support, instrumental support, self-distraction, denial, venting, substance use, behavioral disengagement, and self-blame (40). Items are rated on a 4-point Likert scale from 1 (I haven’t been doing this at all) to 4 (I’ve been doing this a lot). Each coping strategy score represents the sum of its two items.

In this study, coping strategies were analyzed using a two-factor classification system. They were grouped into adaptive coping (active coping, instrumental support, planning, emotional support, positive reframing, humor, acceptance, religion) and maladaptive coping (self-distraction, denial, substance use, behavioral disengagement, venting, self-blame). Subscale scores represent the sum of their constituent coping strategy scores.

The Brief COPE has demonstrated acceptable reliability across diverse populations, with internal consistency coefficients typically ranging from 0.50 to 0.90 for individual subscales (40). For our sample, it demonstrated acceptable internal consistence (Cronbach’s alpha *α* = 0.78).

#### 2.2.4 THINC-it^®^ cognitive screener

The THINC-it^®^ cognitive screener is a brief computerized cognitive screening tool designed to evaluate cognitive functioning, and it has been previously validated in individuals diagnosed with MDD (41). For our study, the THINC-it^®^ was administered as a desktop application and comprises four objective, previously-validated cognitive tests including Spotter (Choice Reaction Time, CRT), Symbol Check (the 1-back version of the N-back working memory test), Codebreaker (Digit Symbol Substitution Test, DSST), and Trails (Trail Making Test-B, TMT-B). It also includes a self-reported cognitive function questionnaire – the 5-item variant of the Perceived Deficits Questionnaire - Depression (PDQ-D-5). In total, the four tests and the questionnaire take about 10 minutes to complete.

Spotter measures attention and processing speed by requiring participants to respond to visual stimuli as quickly and accurately as possible. Symbol Check assesses working memory by asking participants to identify when a current stimulus matches the previous one. Codebreaker evaluates processing speed and attention through a symbol-digit matching task. Trails measures executive function and cognitive flexibility by requiring participants to connect numbered and lettered circles in alternating sequence. The PDQ-D-5 is a 5-item self-report measure assessing perceived cognitive difficulties (also referred to as perceived cognitive deficits) in daily functioning.

Outcome measures included number of correct responses and reaction times (in milliseconds) for CRT and 1-back tasks, number of correct responses for DSST, completion time (in seconds) and number of errors for TMT-B, and total score for PDQ-D-5. Higher scores on the objective cognitive tests (except reaction times and completion times) indicate better performance, while higher PDQ-D-5 scores indicate greater perceived cognitive deficits. The PDQ-D-5 demonstrated good internal consistency in this sample (Cronbach’s alpha *α* = 0.86).

### 2.3 Statistical analyses

All analyses were conducted using R version 4.2.3 and Python version 3.11 with standard statistical packages. Two-tailed tests were applied throughout with a significance threshold of *α* = 0.05. To account for multiple testing, the Benjamini–Hochberg false discovery rate (FDR) approach (42) was applied.

Group comparisons were performed using independent-samples *t*-tests for continuous variables, with Welch’s correction applied when variances were unequal, and Pearson’s χ² tests or Fisher’s exact tests for categorical variables. Within each analytic domain (demographic, clinical, personality, coping, cognition), *p*-values were FDR-adjusted. Both uncorrected *p*-values and FDR-adjusted *q*-values are reported, with *q* < 0.05 considered statistically significant. Effect sizes are presented as Cohen’s *d* for continuous variables and odds ratios (*ORs*) with 95% confidence intervals (*CIs*) for categorical variables.

Associations among clinical, personality, coping, cognitive, and demographic variables were examined using Spearman’s rank-order correlations, with FDR correction applied within and between domains. Hierarchical logistic regression was used to identify predictors of MDD status (binary classification: individual diagnosed with MDD versus healthy control), with predictors entered in blocks (demographics → personality → coping → cognition) and the order reflecting the layered model of depression vulnerability outlined earlier in the Introduction. Following established guidelines recommending at least 10 events per predictor to minimize overfitting and ensure stable parameter estimates (43, 44), and given 36 participants with MDD, the model was restricted to four predictors. Model performance was evaluated using the area under the receiver operating characteristic curve (*AUC*) with 95% confidence intervals, and incremental predictive value was assessed using likelihood ratio tests comparing nested models.

Hierarchical linear regression was also conducted within the patient subgroup to examine predictors of depression severity (HAM-D-17 scores). Predictors were entered in the same blocks as above, and model performance was assessed using *R²*, adjusted *R²*, Δ*R²*, *F*-tests, and standardized effect sizes (Cohen’s *f²*).

## 3 Results

### 3.1 Participant characteristics and group comparisons

Descriptive statistics — including demographics, clinical characteristics, personality, coping styles, and cognition — and the differences between patient and control group are presented in **Table 1**.

**Table 1:** Group comparisons between patients and healthy controls.

Domain	Variable	Patients (*N* = 36)	Healthy controls (*N* = 36)	*p* value	*q* value	Effect size
Demographics	***Sex***			0.806	0.984	
	Male	14 (38.9%)	12 (33.3%)			
	Female	22 (61.1%)	24 (66.7%)			
	**Age (years)**	24.43 ± 2.71	24.42 ± 2.24	0.984	0.984	0.01
	***Ethnicity***			0.791	0.984	
	Chinese	28 (77.8%)	30 (83.4%)			
	Malay	6 (16.7%)	4 (11.1%)			
	Others	2 (5.5%)	2 (5.5%)			
	***Highest education level***			< 0.001	**< 0.001**	
	Degree	15 (41.7%)	33 (91.7%)			
	Diploma	17 (47.2%)	3 (8.3%)			
	GCE ‘A’ Level and below	4 (11.1%)	0 (0.0%)			
Clinical	HAM-D-17 total score	15.61 ± 6.33	1.89 ± 2.33	< 0.001	**< 0.001**	**3.00**
	HAM-D-17 insomnia domain score	1.83 ± 1.65	0.28 ± 0.57	< 0.001	**< 0.001**	**1.33**
	HAM-D-17 anxiety domain score	3.39 ± 2.28	0.47 ± 0.91	< 0.001	**< 0.001**	**1.81**
	HAM-D-17 somatic domain score	1.75 ± 1.13	0.03 ± 0.17	< 0.001	**< 0.001**	**1.62**
	HAM-D-17 melancholia domain score	6.97 ± 3.23	1.19 ± 1.56	< 0.001	**< 0.001**	**2.02**
	HAM-D-17 response-based domain score	7.61 ± 3.73	1.22 ± 1.61	< 0.001	**< 0.001**	**1.91**
Personality	Extraversion score	2.67 ± 1.28	4.08 ± 1.47	< 0.001	**< 0.001**	**-0.99**
	Agreeableness score	4.40 ± 1.50	5.26 ± 1.03	0.006	**0.008**	**-0.61**
	Conscientiousness score	3.81 ± 1.65	4.74 ± 1.12	0.007	**0.008**	**-0.66**
	Emotional stability score	2.60 ± 1.05	4.69 ± 1.37	< 0.001	**< 0.001**	**-1.72**
	Open-mindedness score	4.54 ± 1.22	4.68 ± 1.20	0.627	0.627	-0.11
Coping styles	Adaptive coping	37.39 ± 7.65	42.69 ± 9.20	0.010	**0.010**	**-0.63**
	Maladaptive coping	26.58 ± 4.78	22.58 ± 5.24	0.001	**0.002**	**0.80**
Cognition	***Attention* sp*eed***					
	CRT – Number of correct responses	38.67 ± 1.43	39.08 ± 1.27	0.197	0.284	-0.30
	CRT - Reaction time (ms)	430.84 ± 128.95	458.15 ± 124.85	0.364	0.474	-0.22
	***Working memory***					
	N-Back – Number of correct responses	22.97 ± 10.19	23.11 ± 8.85	0.951	0.983	-0.01
	N-Back - Reaction time (ms)	982.59 ± 237.52	917.87 ± 154.77	0.176	0.264	0.29
	***Processing* sp*eed***					
	DSST – Number of correct responses	53.42 ± 16.32	59.22 ± 14.29	0.113	0.176	-0.38
	***Executive function***					
	TMT-B - Completion time (s)	25.17 ± 9.62	22.39 ± 8.84	0.206	0.287	0.30
	TMT-B – Number of errors	0.69 ± 1.01	0.67 ± 0.93	0.904	0.979	0.02
	***Perceived cognitive deficits***					
	PDQ-D-5 total score	13.00 ± 3.67	6.36 ± 3.80	< 0.001	**< 0.001**	**1.78**

Means and standard deviations are reported for continuous variables; counts and percentages are reported for categorical variables. *p* values reflect uncorrected results from independent t-tests and chi-square tests. *q* values represent FDR-adjusted *p* values using the Benjamini–Hochberg procedure. Effect sizes are reported as Cohen’s *d* for continuous variables.

Bold values indicate statistical significance.

Patients differed significantly from HCs in terms of their highest level of education obtained (*q* < 0.001), and had significantly higher HAM-D-17 (*d* = 3.00, *q* < 0.001). In terms of personality, patients also demonstrated pronounced differences from controls, with especially lower emotional stability (*d* = –1.72, *q* < 0.001) and extraversion (*d* = –0.99, *q* < 0.001), alongside lower agreeableness, and conscientiousness (**Table 1**). In the coping styles domain, patients reported reduced use of adaptive strategies (*d* = -0.63, *q* = 0.010) and greater reliance on maladaptive coping strategies (*d* = 0.80, *q* = 0.003). They also reported substantially greater perceived cognitive deficits (*d* = 1.78, *q* < 0.001), despite no significant group differences on objective cognitive task performance across domains.

### 3.2 Associations among clinical, personality, coping, and cognitive variables

**Figure 1:**
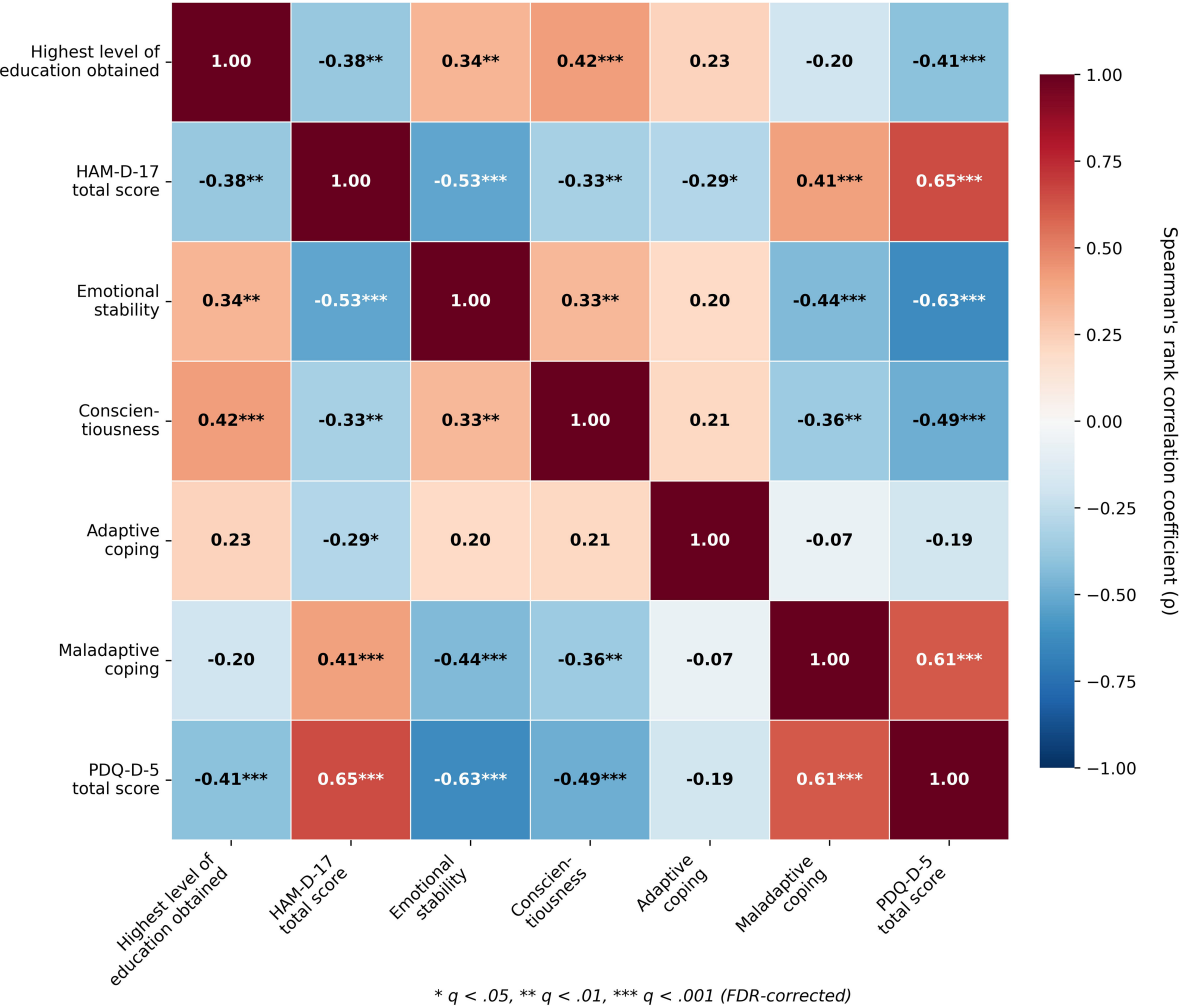
Correlation matrix of depression severity and psychological and cognitive variables. Spearman rank correlation coefficients (ρ) are displayed with FDR-corrected significance indicators: *q < 0.05, **q < 0.01, ***q < 0.001. *Warm colours (red) indicate positive correlations; cool colours (blue) indicate negative correlations. Highest level of education obtained coded as 1 = degree*, *0 = diploma and below.*.

Spearman rank correlation analyses were also conducted to examine broad patterns of association among clinical, personality, coping, cognitive, and demographic variables. Several significant FDR-corrected correlations (*q* < 0.05) were observed (see **Figure 1**).

Depression severity (as measured by HAM-D-17 total score) showed strong positive correlations with perceived cognitive deficits (ρ = 0.65, *q* < 0.001) and moderate correlations with maladaptive coping (ρ = 0.41, *q* < 0.001), and was negatively associated with emotional stability (ρ = -0.54, *q* < 0.001), education (ρ = -0.38, *q* = 0.002), conscientiousness (ρ = -0.33, *q* = 0.007), and adaptive coping (ρ = -0.29, *q* = 0.020).

Among the psychological and cognitive factors, emotional stability showed strong negative correlations with both perceived cognitive deficits (ρ = -0.63, *q* < 0.001) and maladaptive coping (ρ = -0.44, *q* <.001). Education correlated positively with conscientiousness (ρ = 0.42, *q* <.001) and negatively with perceived cognitive deficits (ρ = -0.41, *q* <.001). The strongest correlation that emerged was between maladaptive coping and perceived cognitive deficits (ρ = 0.61, *q* < 0.001).

Given these associations, 4 predictors were selected for hierarchical logistic regression based on: (1) FDR-corrected significance in group comparisons (**Table 1**), (2) effect size magnitude, and (3) theoretical alignment with the layered vulnerabilities framework. From the FDR-significant predictors, the strongest representative from each theoretical layer was selected: highest level of education obtained (demographic), emotional stability (personality), maladaptive coping (coping styles), and perceived cognitive deficits (cognition).

### 3.3 Predictors of depression status

Hierarchical logistic regression was performed to identify predictors of MDD status and to evaluate the incremental diagnostic contribution of personality, coping, and cognitive variables. These predictors were entered sequentially in four blocks: (1) Demographics (highest level of education), (2) personality (emotional stability), (3) coping styles (maladaptive coping), and (4) cognition (perceived cognitive deficits).

**Figure 2:**
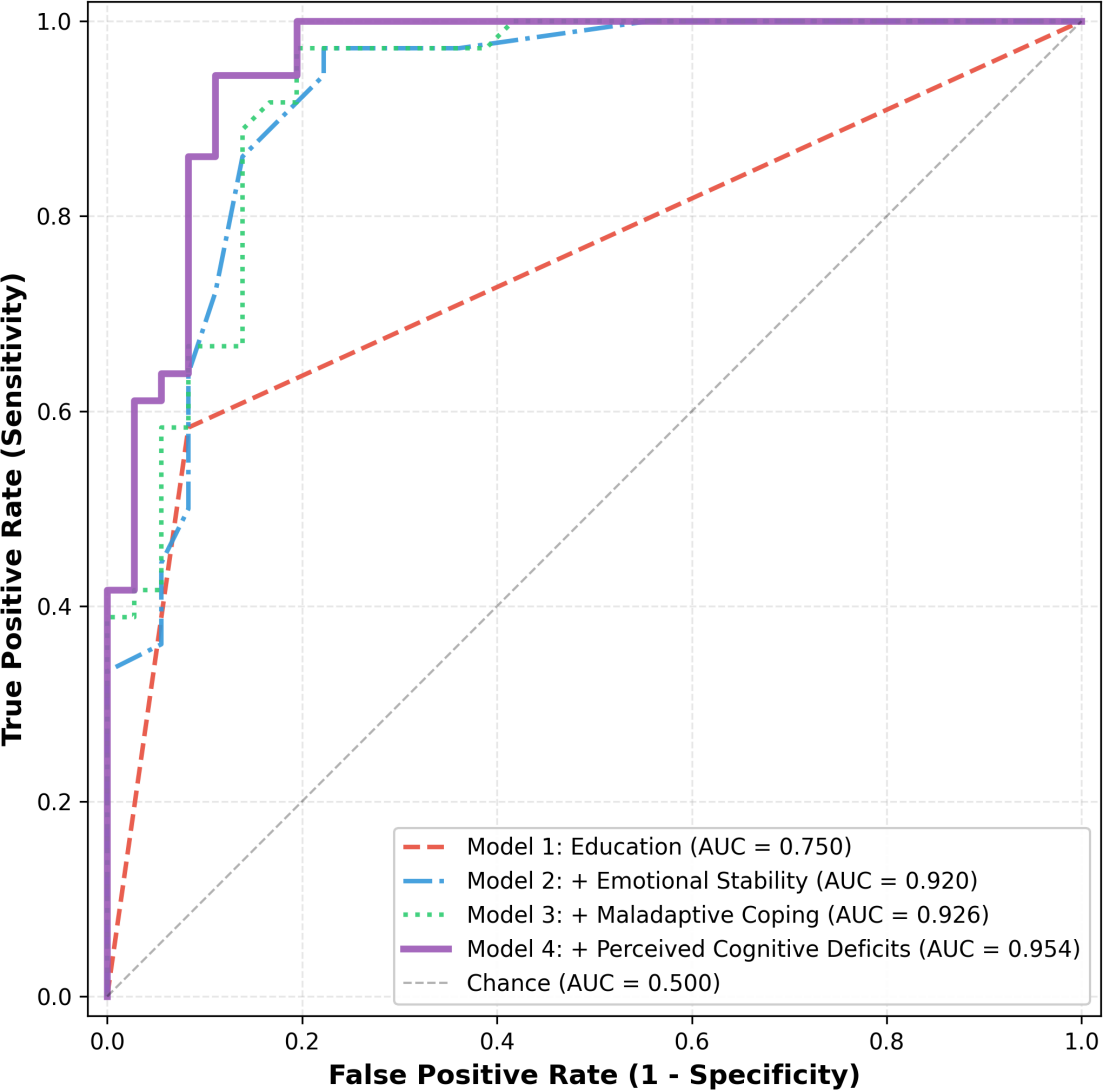
Receiver operating characteristic (ROC) curves showing incremental improvement in the prediction of major depressive disorder (MDD) across hierarchical logistic regression models. Area under the curve (AUC) increased with the addition of each predictor block, demonstrating the incremental value of psychological and cognitive variables in distinguishing MDD cases from healthy controls.

The baseline model including only the highest level of education obtained showed moderate discriminative ability (*AUC* = 0.750, 95% *CI* [0.656, 0.838]; see **Table 2a**). Adding emotional stability markedly improved model performance (*AUC* = 0.920, Δ*AUC* = 0.170, *p* < 0.001). While incorporating maladaptive coping styles did not contribute to a significant increase in *AUC*, the final model incorporating perceived cognitive deficits achieved excellent overall discrimination (*AUC* = 0.954, 95% *CI* [0.910, 0.993]) and further enhanced the previous model (Δ*AUC* = 0.028, *p* = 0.046) (see **Figure 2**).

**Table 2a:** Hierarchical logistic regression models predicting MDD status.

Predictor(s) added	*AUC*	95% *CI*	Δ*AUC*	*p* value
Demographics (highest level of education)	0.750	[0.656, 0.838]	—	—
+ Personality (emotional stability)	0.920	[0.870, 0.975]	0.170	**< 0.001**
+ Coping styles (maladaptive)	0.926	[0.876, 0.979]	0.006	1
+ Cognition (perceived cognitive deficits)	0.954	[0.910, 0.993]	0.028	**0.046**

Model performance (discriminative ability) assessed by area under the receiver operating characteristic (ROC) curve (*AUC)* with 95% *CIs*. ΔAUC represents the change in AUC compared to the previous model, with *p* values reflecting the significance of model improvement based on likelihood ratio tests.

Bold values indicate statistical significance.

In the final hierarchical logistic regression model including four predictors, lower emotional stability and greater perceived cognitive deficits were significantly associated with MDD status. Emotional stability was a significant predictor of MDD status, such that lower emotional stability was associated with higher odds of MDD (OR = 0.409, 95% *CI* [0.184, 0.910], *p* = 0.028; see **Table 2b**). Additionally, greater perceived cognitive deficits significantly increased the odds of being diagnosed with MDD (OR = 1.478, 95% *CI* [1.104, 1.977], *p* = 0.009; see **Table 2b**). In contrast, highest level of education and maladaptive coping were not significant predictors of MDD status.

**Table 2b:** Hierarchical logistic regression models predicting MDD status.

Predictor	*OR*	95% *CI*	*p* value
Demographics			
Highest level of education	0.221	[0.020, 2.417]	0.216
Personality			
Emotional stability	0.409	[0.184, 0.910]	**0.028**
Coping styles			
Maladaptive coping	0.934	[0.779, 1.121]	0.465
Cognition			
Perceived cognitive deficits	1.478	[1.104, 1.977]	**0.009**

Odds ratios (*OR*), 95% confidence intervals (*CI*), and *p* values are reported for each predictor.

Bold values indicate statistical significance.

### 3.4 Predictors of depression severity

Hierarchical linear regression was conducted within the patient group (*N* = 36) to examine whether the same predictors of MDD status also predicted depression severity. Predictors were also entered hierarchically into the regression model in four blocks: (1) Demographics (highest level of education), (2) personality (emotional stability), (3) coping styles (maladaptive coping), and (4) cognition (perceived cognitive deficits). Model performance at each step is summarized in **Table 3a**, showing the incremental variance explained (*R²*, adjusted *R²*, Δ*R²*) and overall model fit statistics (*F*-tests).

**Table 3a:** Hierarchical linear regression models predicting depression severity.

Model	R²	Adjusted R²	ΔR²	*F* statistic	*p* value
Demographics (highest level of education)	0.002	-0.027	—	*F*(1,34) = 0.06	0.801
+ Personality (emotional stability)	0.042	-0.016	0.040	*F*(1,33) = 1.39	0.246
+ Coping styles (maladaptive coping)	0.159	0.080	0.117	*F*(1,32) = 4.45	**0.043**
+ Cognition (perceived cognitive deficits)	0.167	0.060	0.008	*F*(1,31) = 0.29	0.593

R² reflects the proportion of variance in the outcome explained by the model; adjusted R² accounts for model complexity. ΔR² and ΔAdjusted R² represent the change in explained variance after adding each block of predictors. *F* statistics and corresponding *p* values indicate whether the change in model fit is statistically significant.

Bold values indicate statistical significance.

The baseline model with education explained minimal variance in depression severity (R² = 0.002, *p* = 0.801). Adding emotional stability did not significantly improve severity prediction (R² = 0.042, *p* = 0.246). However, the addition of maladaptive coping yielded a significant increase (ΔR² = 0.117, *p* = 0.043), explaining 15.9% of variance. The incorporation of perceived cognitive deficits in the final step did not significantly improve model fit (ΔR² = 0.008, *p* = 0.593), with the final model explaining 16.7% of variance (adjusted R² = 0.060).

**Table 3b** illustrates that none of the individual predictors reached statistical significance in the final hierarchical linear regression model.

**Table 3b:** Hierarchical linear regression models predicting depression severity.

Predictor	B	95% *CI*	β	*p* value
Demographics				
Highest level of education	0.803	[-3.41, 5.02]	0.396	0.712
Personality				
Emotional stability	-0.494	[-2.55, 1.57]	-0.510	0.642
Coping styles				
Maladaptive coping	0.393	[-0.15, 0.94]	1.853	0.169
Cognition				
Perceived cognitive deficits	0.193	[-0.51, 0.89]	0.699	0.593

Unstandardized (B) and standardized (*β*) regression coefficients, 95% CIs, and *p* values are reported for each predictor.

This is likely to be a result of the limited statistical power associated with the small sample size (N = 36), and suggests that while maladaptive coping significantly contributes to predicting MDD severity as part of the hierarchical model (as seen in **Table 3a**), its unique effect after controlling for the other predictors is modest.

## 4 Discussion

Taken together, our findings support a layered model of depression vulnerability in young adults aged 21–29 years. At the distal trait level, lower emotional stability emerged as the most robust personality predictor, demonstrating both large effect sizes in group comparisons and significant predictive utility for MDD status. At the intermediate coping level, patients consistently exhibited greater reliance on maladaptive strategies. Most critically, at the proximal cognitive level, perceived deficits rather than objective cognitive performance showed the strongest association with MDD status, providing remarkable incremental diagnostic utility that elevated the model’s discriminative capacity to an outstanding level. Correlation analyses revealed that these vulnerability dimensions were interconnected, with perceived cognitive deficits showing particularly strong associations with both emotional stability and maladaptive coping, supporting a network model in which distal traits, intermediate coping processes, and proximal cognitive experiences mutually influence one another. These findings extend empirically established associations to a young Singaporean adult sample and support a layered framework in which personality, coping, and cognition interact to shape depression vulnerability.

Our results emphasize lower emotional stability as a distal vulnerability. Depressed YAs scored significantly lower in emotional stability, reflecting higher neuroticism, than HCs, consistent with meta-analyses showing neuroticism as a robust predictor of MDD onset, recurrence, and chronicity (14, 45). Developmentally, neuroticism peaks around age 20, coinciding with heightened vulnerability during this transitional period (2, 15, 20). Depressive episodes may further entrench these tendencies through “scarring effects”, reinforcing dysfunctional emotion regulation patterns (46). Beyond neuroticism, lower conscientiousness, extraversion, and agreeableness were observed, corroborating with evidence linking these traits to impaired self-regulation, diminished social support, and increased sensitivity to environmental stressors (14, 47).

Importantly, adding emotional stability to the baseline (demographic) hierarchical logistic regression model yielded a substantial improvement in diagnostic accuracy, highlighting its utility as a distal predictor of MDD status. This finding aligns with meta-analytic evidence that trait neuroticism is among the strongest personality predictors of depression and improves diagnostic classification beyond demographic factors (14). Moreover, neuroticism may interact with coping and cognitive processes to shape symptom expression, functioning as a transdiagnostic vulnerability that amplifies stress reactivity and maladaptive responses (48). Our previous work identified lower emotional stability as a salient marker of MDD in Singaporean adults (16), and the current study extends this to young Singaporean adults, situating personality within a layered framework alongside coping and cognition. Collectively, these findings underscore emotional stability as both a developmentally and clinically meaningful target for early identification and preventive interventions.

Coping styles also differentiated YAs with MDD from controls, with greater reliance on denial, venting, and self-blame, and reduced use of problem-solving and positive reframing. In our sample, maladaptive coping scores were substantially higher and adaptive coping scores lower among YAs with MDD, consistent with prior evidence that maladaptive coping sustains negative affect through avoidance and rumination, whereas adaptive coping fosters resilience (49, 50). Emerging adulthood is a critical period for shaping coping tendencies, and maladaptive strategies adopted during this time may become entrenched, contributing to long-term vulnerability to MDD (51). In a Singaporean student sample, ‘avoidance’ or ‘wishful thinking’-oriented coping were reported to be less effective at managing stress (52). Maladaptive coping strategies have also been linked to higher levels of psychological distress in a Malaysian sample (53).

Within our sample, adaptive coping showed a modest protective effect against depression severity, while maladaptive coping demonstrated a positive correlation. This aligns with Nolen-Hoeksema’s (2008) theoretical framework that coping styles, rather than stable personality traits, serve as more proximal and malleable determinants of ongoing symptom severity once depression develops (22, 54). The malleability of coping strategies relative to trait-based characteristics positions them as optimal intervention targets, with cognitive-behavioral approaches demonstrating consistent efficacy in modifying maladaptive coping patterns and reducing depressive symptoms (32, 55). Exploratory hierarchical linear regression in the patient subgroup further suggested that maladaptive coping contributed to depressive symptom severity, although the small sample size limits the confidence of this finding.

The most notable finding in our study was the divergence between subjective and objective cognition, with perceived cognitive deficits showing a robust independent association with MDD. Young adults with MDD reported pronounced difficulties in memory, concentration, and decision-making, despite minimal differences from healthy controls on their objective performance on the 4 cognitive tests. This aligns with international evidence that subjective cognitive complaints track depressive symptoms, functional outcomes, and treatment response more closely than objective performance (27, 28, 56). Subjective cognitive complaints may be particularly salient in young adults, where academic performance, occupational competence, and self-confidence are central to daily functioning (57). Csábi et al. (2025) found subjective complaints predicted functional impairment in students in Hong Kong even without measurable objective deficits, while Srisurapanont et al. (2018) observed stronger subjective complaints among younger depressed patients. Sleep disruption and stress further exacerbate these complaints, underscoring their modifiable and clinically relevant nature (23, 58).

Within our layered framework, subjective cognition represents the most proximal, state-dependent layer of vulnerability, directly capturing the lived experience of young adults with depression. Perceived cognitive deficits contributed meaningful incremental predictive value in the hierarchical logistic regression model, capturing aspects of depression vulnerability not fully accounted for by personality or coping. This corroborates existing evidence that self-reported cognitive complaints better reflect depressive severity, functional impairment, and quality of life than objective tests (56, 59, 60). Brief instruments such as the PDQ-D (and its variants) can therefore enhance feasibility and acceptability in high-throughput settings without compromising diagnostic precision, providing a practical and sensitive means of identifying young adults at elevated risk for depression (28, 61).

Coping styles and subjective cognitive complaints showed to be most strongly correlated with depression severity, whereas personality traits played a lesser role once MDD was established. This supports layered vulnerability models, where distal traits confer background risk, but more state-like processes such as coping and subjective cognition track symptom burden more closely (34, 48). Cross-domain associations, where low emotional stability was associated with maladaptive coping and more perceived cognitive deficits, align with depression network models in which neuroticism heightens stress reactivity, maladaptive coping maintains distress, and negative self-appraisals reinforce impairment (5, 62). For young adults navigating academic and social transitions, this interplay may create a self-sustaining cycle of distress. Interventions that disrupt these links, such as coping flexibility or metacognitive therapies, may reduce underlying vulnerability (63, 64). Education also showed significant negative associations with both depression severity and perceived cognitive deficits at the univariate level, though it did not emerge as an independent predictor in the multivariate model. This aligns with evidence that education’s protective effects against depression operate primarily through psychosocial pathways such as enhanced coping resources and cognitive flexibility (65), rather than as a direct, independent protective factor.

The strengths of this study include its focus on young adults aged 21–29 and its multidimensional assessment of personality, coping, and cognition, which provided a more holistic perspective on MDD than studies examining single domains in isolation. By evaluating personality traits, coping styles, and both subjective and objective cognition within the same framework, it captured layered vulnerabilities that more closely mirror the real-world complexity of depression. To our knowledge, it is also among the first to explore these domains jointly in young adults, thereby helping to address an important gap in the literature. The use of validated instruments, hierarchical regression, ROC analyses, and false discovery rate correction further enhances methodological rigor and minimizes type I error.

Several limitations should also be acknowledged. The cross-sectional design precluded causal inference, and reliance on self-report measures, particularly for perceived cognitive deficits, may have introduced mood-congruent biases. The small sample size also limited statistical power in regression analyses. Recruitment from outpatient clinics at a single hospital also limited result generalizability. We used brief self-report measures to reduce participant burden, particularly for MDD-diagnosed patients; while these instruments have acceptable reliability, their brevity may result in slightly lower internal consistency compared with longer measures. Similarly, the THINC-it^®^ screener, though validated, may lack sensitivity to subtle objective deficits due to its brevity, particularly in high-functioning MDD patients. While our study was conducted in a young Singaporean adult sample, we did not directly measure cultural variables or perceptions, limiting our ability to examine cultural influences on depression vulnerability profiles in this sample. Future work employing matched cross-cultural designs (i.e., addition of a Western comparator group) would clarify whether personality, coping, and cognitive vulnerabilities operate similarly across cultural contexts or are moderated by cultural factors.

Despite these limitations, our findings carry important implications for diagnosis and treatment. Lower emotional stability and perceived cognitive deficits emerged as the strongest predictors of MDD status, underscoring the utility of brief self-report tools such as the PDQ-D-5 as practical frontline screeners in both primary care and mental health services. Unlike extensive neurocognitive batteries, these tools can be administered quickly, require minimal resources, and are more acceptable to young adults who may underreport mood symptoms (35, 66). While stigma around mental health affects help-seeking globally, the specific ways young adults express psychological distress may vary across cultural contexts. Some evidence suggests that somatic and cognitive complaints may be particularly salient presentation patterns in Asian clinical settings (51, 67), though our study did not directly examine these cultural factors. Regardless of cultural context, brief tools assessing perceived cognitive deficits offer a practical, acceptable screening approach for young adults who may underreport mood symptoms.

The layered framework adopted in this study also provides a structured roadmap for intervention. At the distal level, personality-based vulnerabilities such as low emotional stability may be addressed through early intervention programs targeting emotion regulation and resilience (15, 55). Coping, as a semi-malleable process, would respond to psychosocial and mindfulness-based programs (22, 23). Cognitive behavioral therapy (CBT) has demonstrated significant efficacy in modifying maladaptive coping patterns and enhancing adaptive strategies, thereby reducing depressive symptoms in young adults (32). At a proximal level, perceived cognitive deficits are clinically salient and modifiable. CBT and metacognitive therapies specifically target negative self-appraisals and maladaptive thinking patterns, while lifestyle interventions such as sleep optimization and stress reduction address contextual factors that exacerbate perceived cognitive difficulties. By targeting these proximal, state-like complaints, such approaches can meaningfully reduce subjective cognitive burden and improve daily functioning, even when objective performance remains largely intact (31, 58). Together, these strategies offer clear intervention targets across distal, intermediate, and proximal levels, supporting personalized, developmentally-relevant care and highlighting modifiable pathways to reduce vulnerability and improve outcomes in young adults with depression.

In conclusion, this study demonstrates that low emotional stability and subjective cognitive complaints and, to a lesser extent, coping styles, constitute layered vulnerabilities for major depressive disorder in young adults. While traits provide distal predispositions, subjective cognition serves as a proximal, state-dependent marker that strongly enhances diagnostic accuracy. Extending established findings to a young Singaporean adult sample, these results underscore the clinical and public health value of moving beyond symptom-based models toward multidimensional, developmentally sensitive approaches. By highlighting both risk profiling and modifiable targets, this work supports more precise strategies for the early detection and management of depression in young adults.

## Data Availability

The raw data supporting the conclusions of this article will be made available by the corresponding, without undue reservation.
